# Risk Factors Associated With the Development of Acute Peripancreatic Fluid Collections on Follow-Up Imaging After Acute Pancreatitis: What Physicians Need to Know

**DOI:** 10.7759/cureus.50471

**Published:** 2023-12-13

**Authors:** Nouran W Molla, Renad H Zaini, Fahad A Alfaiz, Ahmad M Alkhayatt, Majed A AlJohani, Mohammed O Alomar, Abdulaziz A Aljohani, Mohammed S BinMayouf, Abduljabbar A Alyamani, Abdullah H Alsergani

**Affiliations:** 1 Radiology, King Saud University, Riyadh, SAU; 2 Medical Education, Princess Nourah Bint Abdul Rahman University, Riyadh, SAU; 3 Radiology, King Faisal Specialist Hospital and Research Centre, Riyadh, SAU; 4 Medical Education, King Saud University, Riyadh, SAU; 5 Neurology, King Faisal Specialist Hospital and Research Centre, Riyadh, SAU; 6 Internal Medicine, King Khalid University Hospital, Riyadh, SAU; 7 Family Medicine, King Saud University, Riyadh, SAU; 8 Dermatology, King Fahad Medical City, Riyadh, SAU; 9 Otolaryngology - Head and Neck Surgery, King Faisal Specialist Hospital and Research Centre, Riyadh, SAU

**Keywords:** predictors, pancreatitis, radiology, apfc, incidence, retrospective, risk factors, follow-up imaging, acute peripancreatic fluid collections, acute pancreatitis

## Abstract

Objectives: This study aims to identify various risk factors for acute peripancreatic fluid collections (APFCs) in patients presenting with acute pancreatitis (AP).

Methods: A blinded retrospective case-control study was conducted at a tertiary care hospital in Riyadh. Data from 327 patients who presented with AP between January 2008 and 2021 were analyzed. Following the application of inclusion/exclusion criteria, the final sample size consisted of 82 patients. Patients were divided into cases and controls based on the presence or absence of APFCs, respectively. APFCs were defined as fluid collections in the peripancreatic region that develop within four weeks of presentation without well-defined walls or solid internal components. Demographic, clinical, and laboratory variables were collected and subjected to multivariate binary regression analysis to assess the odds of developing APFCs.

Results: A total of 34 patients were categorized as cases, while 48 patients were controls. A significant association was found between age (P=0.022), total bilirubin (P=0.012), lipase level (P<0.001), albumin level (P=0.038), and lactate dehydrogenase (LDH) (P=0.037) on admission and the odds of developing APFCs.

Conclusion: Older age, higher levels of bilirubin and lipase, and low levels of albumin and LDH were found to be risk factors for developing APFCs. No other variables were found to be significant. The findings of this study may provide insight into how often clinicians can expect APFCs in patients presenting with AP.

## Introduction

Acute pancreatitis (AP) is a potentially reversible condition in which pancreatic tissue becomes inflamed [1]; the diagnostic criteria are met when at least two of the following three conditions are fulfilled: abdominal pain consistent with AP, serum amylase or lipase levels exceeding three times the upper normal limit, and imaging results indicative of AP [2]. In 80% of patients, AP is mild and self-resolving without significant issues; however, complications arise in up to 20% of patients, leading to substantial mortality rates [3].

Though the origins of AP are often complex and multifactorial, alcohol abuse and biliary stone diseases are the most common causative etiologies [4]. AP can also arise as a result of complications after procedures such as endoscopic retrograde cholangiopancreatography (ERCP), pancreatic surgery, or biopsy [4]. Complications of AP include acute peripancreatic fluid collections (APFCs), pancreatic pseudocysts, acute necrotic collections, and walled-off necroses. APFCs occur around the pancreatic region without well-defined walls or solid internal components [5]; they typically develop within four weeks of AP. Radiological imaging through CT, MRI, and ultrasound is an important part of AP diagnosis, follow-up, and elimination of the aforementioned local complications as possibilities [6]. Although most APFCs are mild and resolve spontaneously without any intervention, management is usually recommended in symptomatic cases and those complicated by infection and sepsis [7]. APFCs are usually then usually drained endoscopically or, in more severe cases, managed surgically [7]. According to the findings in previous studies, the development of peripancreatic fluid collection is an important prognostic indicator of the severity of AP [8,9]. The incidence of APFCs in patients with AP is between 30% and 60%, according to the literature [10]. The clinical significance of APFCs has led to many studies in which researchers investigate potential risk factors associated with their development. An alcoholic etiology of AP, the presence of ascites, older age, and high C-reactive protein levels are significantly associated with the development of APFCs, as concluded in multiple studies [10-12].

This study is a follow-up of a previous study by the same group of authors, the aim of which was to calculate the incidence of APFCs in patients presenting with AP; the incidence rate found in the study was 48% in Saudi populations [6]. This study was performed on the same patient population with a few alterations in the inclusion and exclusion criteria, which resulted in an entirely different sample. The objective of this study is to identify possible risk factors that may predict the development of APFCs. Not many studies have investigated this specific complication of AP in the literature. The significance of this study lies in the fact that since APFCs have been found to predict increased severity and morbidity over the course of AP [8], finding methods to predict its occurrence may help limit the escalation of severity in select cases.

## Materials and methods

Study design

This blinded retrospective case-control study was conducted at a tertiary care center in Riyadh, Saudi Arabia. The patients’ data was collected using the electronic medical records (EMR) system and the radiology information system (RIS). The inclusion criteria of this study were as follows: (i) patients aged above 18 years; (ii) patients diagnosed with AP; (iii) availability of demographic, clinical, and laboratory information pertinent to the study variables; and (iv) existence of follow-up imaging within four weeks of admission in which the presence or absence of APFCs was indicated. The exclusion criterion was the absence of data included in the study's variables. Cases were characterized as patients who developed APFCs, while controls were those who did not.

Data collection

**Figure 1:**
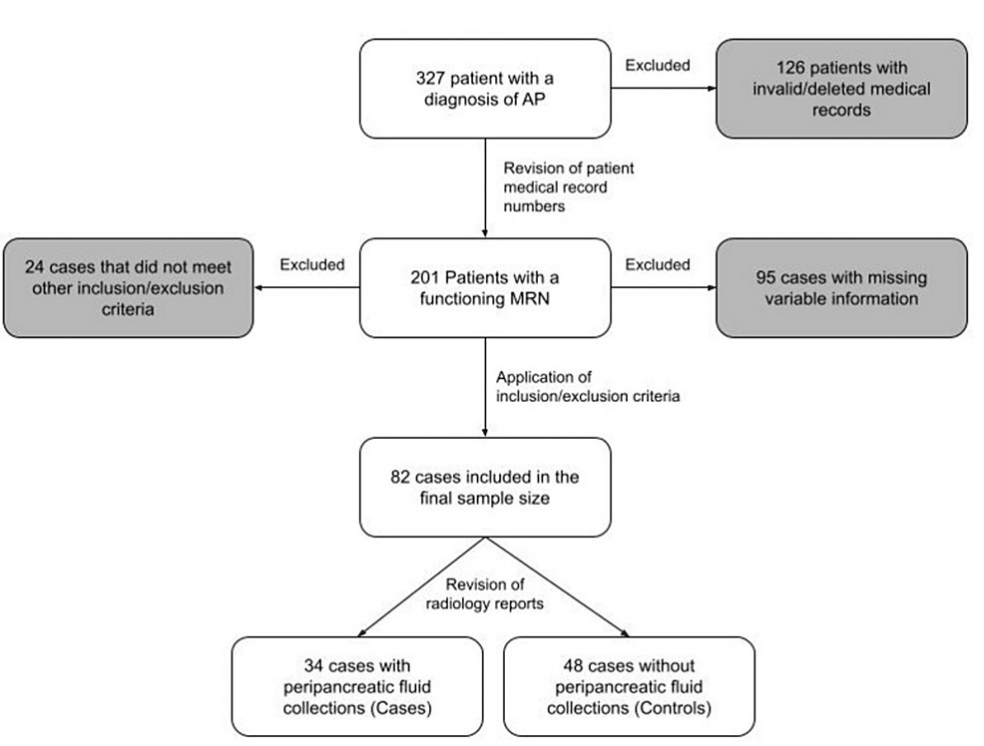
Flowchart demonstrating the data collection process

The study’s population included 327 patients who were collected and sorted in the previous study, comprising patients who were admitted to the hospital for AP between January 2008 and January 2021. Applying the inclusion and exclusion criteria produced a final sample of 82 patients. The significant reduction in sample size was primarily due to the exclusion of patients whose charts lacked study-pertinent clinical and laboratory data. The data collection process is illustrated in greater detail in Figure 1. After obtaining approval from the hospital's IRB for this project, the Institutional Review Board of King Saud University - College of Medicine (approval number: E-22-6585), data regarding the etiology of AP and the presence or absence of APFCs was collected using the hospital’s EMR and follow-up imaging that was conducted within four weeks of patient presentation; however, these details were hidden from the authors before they initiated the data collection process. The demographic and clinical variables gathered from the patients included age, gender, nationality, etiology of pancreatitis, and the presence or absence of diabetes, hypertension, hyperlipidemia, and liver cirrhosis, whereas the laboratory variables were divided and collected in two intervals: upon admission and 48 hours after admission. These variables included WBC count, hematocrit (HCT), alanine transaminase (ALT), aspartate transaminase (AST), alkaline phosphatase (ALP), albumin, creatinine, total bilirubin, gamma-glutamyl transferase (GGT), amylase, lipase, and lactate dehydrogenase (LDH).

Statistical analysis

The mean and standard deviation (SD) were used to describe continuous variables, although continuous variables with evidence of statistical skewness were described with the median and interquartile ranges. Frequencies and percentages were used to describe categorically measured variables. A multiple-response dichotomy analysis was employed to explain variables that were measured with multiple options (“choose all that apply” questions for comorbid conditions, for example). Histograms and the statistical Kolmogorov-Smirnov test of normality were used to assess the statistical normality assumption of metric variables. A multivariable binary logistic regression analysis was used to assess the statistical significance of the risk factors for the sample’s odds of developing APFCs. The association between independent risk factors’ variables and the dependent outcome variables analyzed in logistic regression was expressed as multivariable-adjusted odds ratios (ORs), as were the associated 95% confidence intervals. SPSS Statistics version 28 (IBM Corp. Released 2021. IBM SPSS Statistics for Windows, Version 28.0. Armonk, NY: IBM Corp) was used to analyze the statistical data; the alpha significance level was considered at the 0.05 level.

## Results

**Figure 2:**
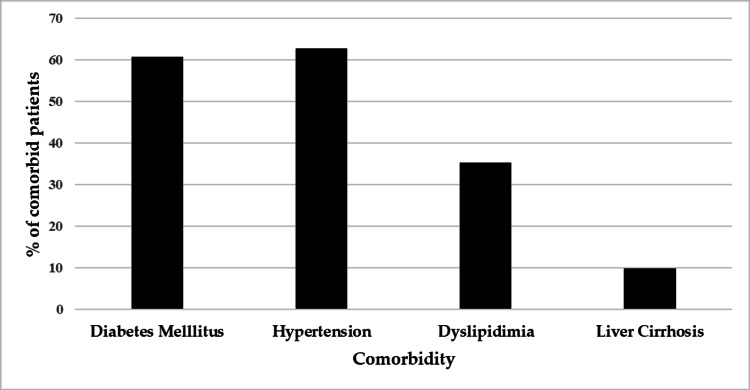
Distribution of patient’s comorbidities

The medical records of 82 patients with AP were reviewed retrospectively; all patients presented with AP between 2008 and 2021. Table 1 displays the descriptive analysis of the patient’s sociodemographic characteristics and past medical histories. The analysis revealed that 58.5% (N=48) of the patients were male and 41.5% were female (N=34). The mean ± SD age of the sample was 49.89 ± 16.83 years, and the majority were Saudi citizens (90.2%, N=74). The most common etiology of pancreatitis was idiopathic (79.3%, N=65), which was followed by biliary causes (14.6%, N=12), ERCP-related causes (2.4%, N=12), and other (3.7%, N=3) causes. Additionally, 62.2% (N=51) of patients suffered from comorbidities, which, as illustrated in Figure 2, were distributed as follows: 62.7% (N=32) had hypertension, 60.8% (N=31) had diabetes, 35.3% (N=18) had dyslipidemia, and 9.8% (N=5) had liver cirrhosis. According to the patient's radiological findings, 41.8% developed APFCs and were subsequently categorized as cases (N=34).

**Table 1:** Demographic data of patients presenting with AP ERCP: endoscopic retrograde cholangiopancreatography

	Frequency	Percentage
Gender		
Male	48	58.5
Female	34	41.5
Age (years), mean±SD		
Age group		
≥46 years	47	57.3
36-45 years	20	24.4
26-35 years	8	9.8
≤25 years	7	8.5
Nationality		
Saudi	74	90.2
Non-Saudi	8	9.8
Primary cause of pancreatitis		
Idiopathic	65	79.3
Biliary	12	14.6
ERCP	2	2.4
Other	3	3.7
Comorbidity		
Yes	51	62.2
No	31	37.8
Comorbidity type		
Hypertension	32	62.7
Diabetes mellitus	31	60.8
Dyslipidemia	18	35.3
Liver cirrhosis	5	9.8
Developed peri-pancreatic fluid collection during admission		
Yes	34	41.5
No	48	58.5

**Figure 3:**
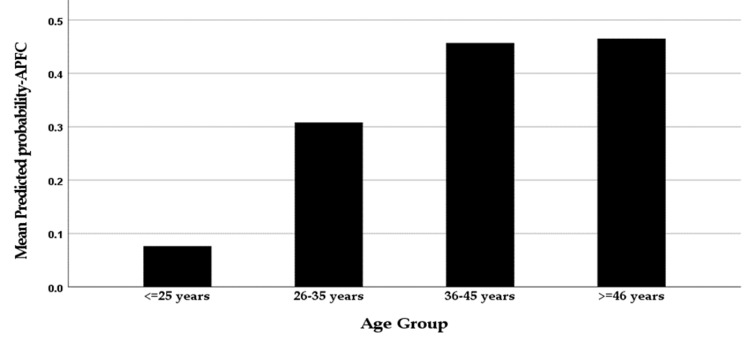
Illustration of the graded association between age and developing APFCs

A multivariable binary logistic regression analysis was applied to determine the patient’s likelihood of developing APFCs. The resulting model, as presented in Table 2, suggests that although gender did not correlate significantly with the odds of developing APFCs (p=0.208), age was significantly correlated (p=0.022). On average, patients aged ≥36 years were significantly more likely (8.101 times more likely) to develop APFCs compared to those aged <36 years. Figure 3 illustrates that patients aged 36-45 years and those aged ≥46 years had a significantly higher mean model-predicted probability of developing APFCs compared to those in younger age groups. The analysis model also indicated that patients with a positive medical history of diabetes and dyslipidemia did not have higher odds of developing APFCs. Concerning lab values, total blood bilirubin and lipase levels on admission correlated significantly and positively with the likelihood of developing APFCs; each additional unit rise in bilirubin level increased the probability of developing APFCs by 2.9 times, on average (p=0.015), while each one-unit increase in serum lipase, on average, raised the odds of developing APFCs by 4.7 times (p<0.001). On the other hand, serum albumin on admission correlated significantly and negatively with the probability of developing APFCs: each additional unit rise in serum albumin levels decreased the odds of developing APFCs by 9.9 times, on average (p=0.038). LDH levels on admission also correlated significantly and negatively with the likelihood of developing APFCs (p=0.037).

**Table 2:** Multivariable logistic binary regression analysis of the patients' odds of developing peripancreatic fluid collection (N=82) Dependent outcome variable: patient developed peripancreatic fluid collection (no/yes). Model overall statistical significance: χ2(9)=33.40, p<0.001. Model Hosmer-Lemeshow chi-square goodness-of-fit index χ2(8)=9.02, p=0.341 LDH: lactate dehydrogenase, OR: odds ratio, CI: confidence interval

	Multivariate adjusted OR	95% CI for OR		p-value
		Lower	Upper	
Gender: Male	2.277	0.632	8.197	0.208
Age group ≥36 years	8.101	1.355	48.439	0.022
Medical history of diabetes mellitus	0.459	0.123	1.717	0.247
Medical history of dyslipidemia	2.276	0.466	11.107	0.309
Admission time serum albumin level	0.901	0.817	0.994	0.038
Admission time blood total blood bilirubin level	1.029	1.005	1.053	0.015
Serum standardized (z-lipase) upon admission time	4.696	2.177	10.127	<0.001
Serum lactic dehydrogenase LDH level on admission	0.989	0.98	0.999	0.037
Primary cause of pancreatitis	1.581	0.679	3.679	0.288
Constant	12.512			0.296

## Discussion

The objective of this study is to determine the risk factors for APFC development in patients presenting with AP. Patients over the age of 46 comprised the majority of the study sample (57.3%), followed by patients between the ages of 36 and 45 (24.4%) and patients below the age of 36 (18.3%). Older age significantly predicts peripancreatic fluid collection, which is consistent with a study in which researchers concluded that age is a significant risk factor for developing all types of AP complications, including fluid collection [13]. Older patient populations are known to experience more severe courses of AP, as indicated by the presence of age on Ranson’s criteria for predicting pancreatitis severity [14]. This may relate to patients developing peripancreatic fluid collections, as this complication has been repeatedly found to be a significant predictor of its severity [9]. Age furthermore contributes to the body’s decrease in physiologic functions, as documented in the literature [13,15]; therefore, older patients are more likely to experience decompensation in the form of said complications.

The results of the analysis also suggest that lower levels of albumin are significantly associated with the development of peripancreatic fluid collection. Low levels of albumin have been consistently correlated with third spacing and intravascular fluid loss, leading to fluid leakage and filtration into the interstitial space [16]. This could further exacerbate the third spacing that is already involved in AP pathophysiology, thus contributing to the development of APFCs. Additionally, a few studies found a direct link between hypoalbuminemia and APFCs. It was found to be associated with an increased incidence of systemic and local complications, including peripancreatic fluid collection, as well as AP severity and mortality [17].

This study also found that bilirubin levels correlated positively and significantly with the patients' odds of developing APFCs, as elevated bilirubin levels implicate biliary system involvement in the pathogenesis of pancreatitis. This could be because of the increased inflammatory pathogenesis of elevated bilirubin levels, which is a known cause of increasing vascular permeability and thus increasing the likelihood of fluid leakage into the extravascular space. The ductal system's participation may also correlate with a more severe course of AP and, thus, an increased disposition for complications such as APFCs [18]. Elevated lipase levels are also believed to be a statistically significant risk factor for APFCs, according to this study’s analysis. The study by Kim et al. [18] further corroborated the findings mentioned prior, as they also concluded that both increased lipase and bilirubin levels, among other biochemical markers taken upon admission, are significantly associated with the radiologic severity of AP according to the Balthazar score, which uses the presence or absence of APFCs as a criterion for pancreatitis severity [18]. That, in turn, helps explain why it was also significantly associated with APFCs in this patient population.

Interestingly, increased LDH levels on admission in this population were significantly and negatively associated with the development of APFCs. This finding contradicts many studies on this topic since LDH is a known risk factor for pancreatitis severity and is part of Ranson's criteria [14]. Cui et al. [10] also indicated that LDH levels 48 hours after admission are significantly associated with peripancreatic fluid collection. A probable explanation for this finding is that patients who have high levels of LDH on admission are more likely to receive increased levels of medical attention since their presentations are more severe. Increased medical attention may limit the incidence of systemic and local complications during the patients' hospital stay, which may have manifested as LDH being protective of APFCs. This could explain why they did not suggest an association with LDH on admission, but after a 48-hour delay, further research on larger patient populations is required to reach a definitive conclusion.

Nevertheless, this study had limitations, such as the relatively small sample size, which may have contributed to lower statistical power, although the issue was addressed by blinding the data collectors to the presence or absence of APFCs, unifying the data collection process, and ensuring that patient data was gathered coherently and completely. Additionally, the poor documentation of alcohol use among patients due to the cultural sensitivity associated with alcohol consumption and the lack of ultrasound studies that confirm biliary etiologies may have contributed to an artificially inflated number of AP cases being listed as idiopathic. Overall, more studies with larger patient samples and more reliable documentation of AP etiology are needed to reach a generalizable conclusion. All in all, the findings of this study may help physicians estimate which patients will develop APFCs and thus be more willing to order follow-up imaging. This may also help physicians be wary of APFCs as a focus of infection should patients develop signs that are suspicious of sepsis. It can also help clinicians more accurately diagnose this complication should patients with a recent history of AP present with vague symptoms that may be suggestive of APFCs, such as abdominal pain. Another possible way they can be used is as evidence of disease severity. This in turn helps physicians with the decision of escalation of care or otherwise.

## Conclusions

APFC is one of many complications that add to the mental, physical, and economic burden of patients with AP. The objective of this study was to investigate risk factors associated with APFC. This study suggests that older age, increased bilirubin and lipase levels, and low levels of albumin and LDH are significantly associated with the likelihood of developing APFCs. The relatively small sample size and the inflated number of idiopathic cases may have contributed to lowering this study’s statistical power, although steps taken while conducting this study’s methodology addressed the issue. Nevertheless, more studies regarding APFCs are needed to reach a definitive conclusion.
